# Dentin dysplasia type I: a challenge for treatment with dental implants

**DOI:** 10.1186/1746-160X-3-31

**Published:** 2007-08-22

**Authors:** Rita A Depprich, Michelle A Ommerborn, Jörg GK Handschel, Christian D Naujoks, Ulrich Meyer, Norbert R Kübler

**Affiliations:** 1 Department for Cranio- and Maxillofacial Surgery, Heinrich-Heine-University Düsseldorf, Moorenstr. 5, 40225 Düsseldorf, Germany; 2 Department for Operative and Preventive Dentistry and Endodontics, Heinrich-Heine-University Düsseldorf, Moorenstr. 5, 40225 Düsseldorf, Germany

## Abstract

**Background:**

Dentin dysplasia type I is characterized by a defect of dentin development with clinical normal appearance of the permanent teeth but no or only rudimentary root formation. Early loss of all teeth and concomitant underdevelopment of the jaws are challenging for successful treatment with dental implants.

**Methods:**

A combination of sinus lifting and onlay bone augmentation based on treatment planning using stereolithographic templates was used in a patient with dentin dysplasia type I to rehabilitate the masticatory function.

**Results:**

(i) a predisposition for an increased and accelerated bone resorption was observed in our patient, (ii) bone augmentation was successful using a mixture of allogenic graft material with autogenous bone preventing fast bone resorption, (iii) surgical planning, based on stereolithographic models and surgical templates, facilitated the accurate placement of dental implants.

**Conclusion:**

Bony augmentation and elaborate treatment planning is helpful for oral rehabilitation of patients with dentin dysplasia type I.

## Background

Dentin dysplasia is a defect of dentin development that is inherited as an autosomal dominant trait and classified into two types [1,2]. Dentin dysplasia type I is characterized by the presence of primary and permanent teeth with normal appearance of the crown but no or only rudimentary root development, incomplete or total obliteration of the pulp chamber and periapical radiolucent areas or cysts. Dentin dysplasia type II is characterized by primary teeth with complete pulpal obliteration and brown or amber bluish coloration similar to that seen in hereditary opalescent dentin. The permanent teeth have a normal appearance or a slight amber coloration, the roots are normal in size and shape with a thistle-tube-shaped pulp chamber with pulp stones [3,4].

The sequelae of dentin dysplasia are difficult to manage and provide a challenge for the dentist concerning restorative and endodontic treatment but also prosthetic treatment after loss of teeth [5]. This report describes the implant based oral rehabilitation of a patient with dentin dysplasia type I including aesthetic considerations, treatment planning using stereolithographic templates and tissue regeneration.

## Case presentation

A 17-year-old girl with a history of dentin dysplasia type I but no other serious diseases, came to our departement for consultation complaining her loose teeth and asking for prosthetic treatment. The girl's mother suffered from the same disease and her edentulous jaws were treated with removable prostheses.

**Figure 1:**
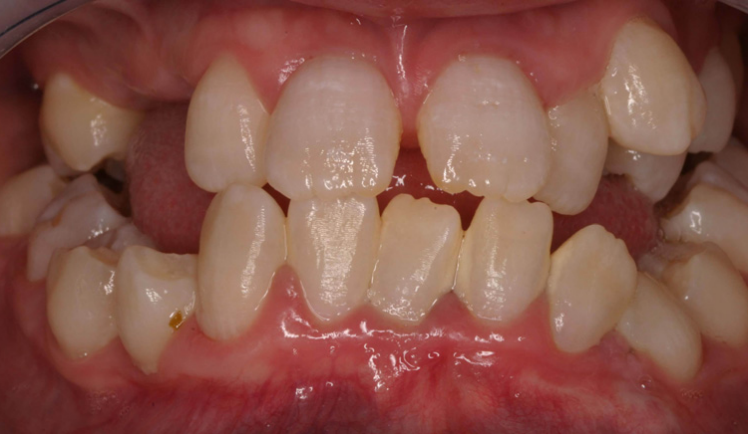
initial clinical situation.

**Figure 2:**
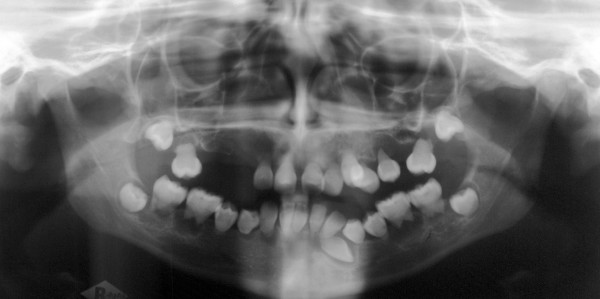
preoperative panoramic radiographs showing features of dentin dysplasia type I.

The clinical examination revealed 2^nd ^to 3^rd ^degree loose permanent teeth normal in shape and size, vertical and sagittal underdevelopment of the maxilla and the mandible, missing teeth 13, 14, 15, 17, 27, 33. The panoramic radiographs showed features characteristic of dentin dysplasia type I with normal appearance of the crown but no root development of all teeth and periapical cysts, in addition to retained teeth 33, 18, 28, 38, 48 (figures 1 and 2).

**Figure 3:**
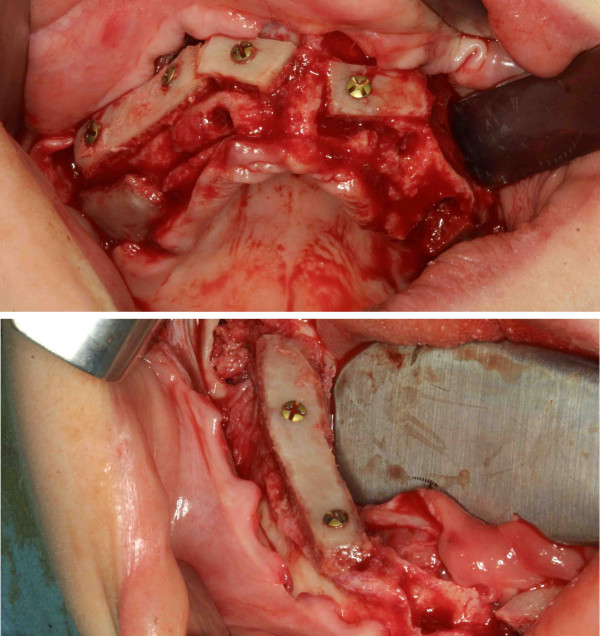
alveolar ridge augmentation of the maxilla (above) and the mandible (below) using autogenous bone grafts from the iliac crest.

**Figure 4:**
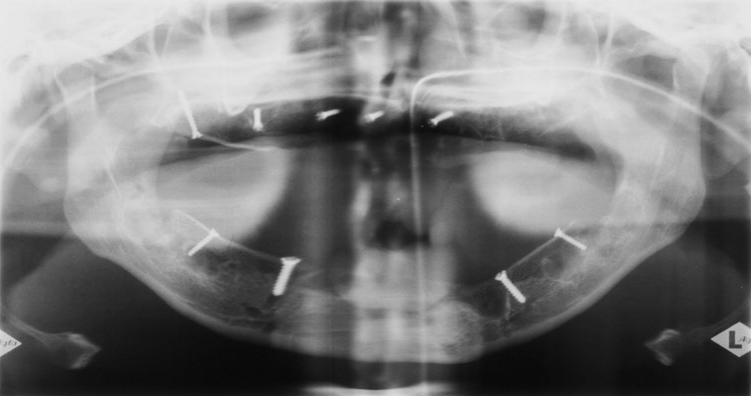
postoperative panoramic radiographs after tooth extraction and bone augmentation.

Initially, extraction of all teeth and cystectomy was performed under general anaesthesia. To reconstitute the lacking bone, a bilateral sinus lifting procedure and a simultaneous alveolar ridge augmentation of the maxilla and the mandible using autogenous corticocancellous block and particulate bone grafts from the iliac crest were peformed (figures 3 and 4). Postoperative healing was uneventful and no dehiscence defect occured.

**Figure 5:**
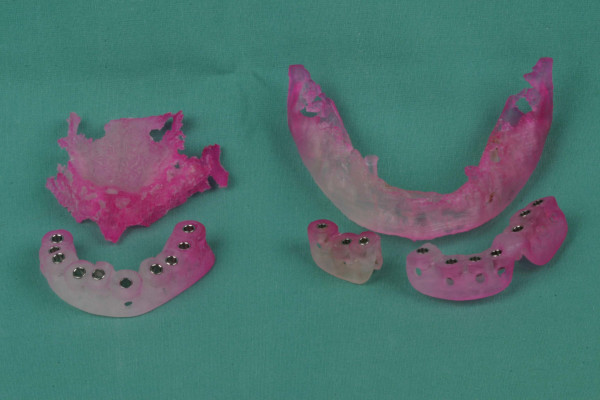
stereolithographic templates with drill-guide tubes manufactured on three-dimensional stereolithographic models of the mandible and maxilla.

Two months later first signs of bone resorption were seen clinically and on the panoramic radiographs. Computed tomography (CT) scan with special scan protheses (mixture of rasin and BaSO4) for implant planning was arranged. The CT scan showed a high degree of resorption of the augmented bone. The digital data from the CT scan were transferred to a personnal computer (PC) and SimPlant^® ^software (Materialise, Leuven, Belgium) was used. Three-dimensional implant planning was performed considering position, angulation and depth of implants in areas of bone augmentation including the aspect of bone density of the augmented bone. Using SurgiGuide^® ^technology (Materialise, Leuven, Belgium) stereolithographic templates containing drill-guiding tubes were manufactured on three-dimensional stereolithographic models of the mandible and maxilla (figure 5).

**Figure 6:**
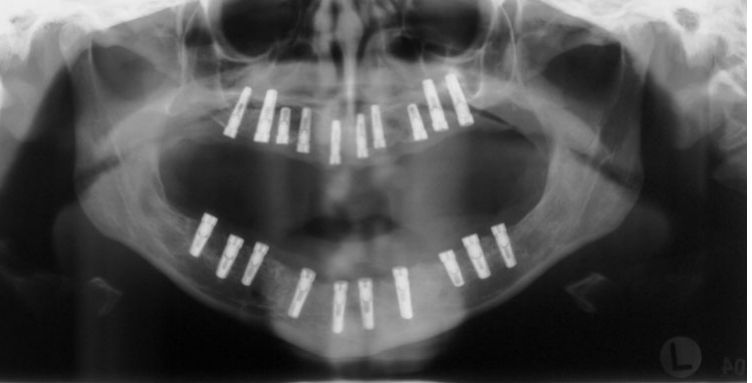
postoperative panoramic radiographs after implant setting and bone augmentation.

After 4 months of socket healing implant surgery was performed under general anaesthesia. The reopening of the mucoperiostal flaps revealed that the augmented bone had been resorbed to a significant extend within four months. By means of the prefabricated templates 10 standard self-tapping implants were inserted in the mandible and the maxilla, respectively, according to the predefined planning (figure 6). Bone augmentation around the dental implants was performed using a mixture (ratio 1:1) of cancellous bone from the iliac crest and Bio-Oss^® ^(particle size 1–2 mm) (Geistlich, Wolhusen, Switzerland) held in place by a bioresorbable collagen membrane (BioMend Extend^®^, Geistlich, Wolhusen, Switzerland). Postoperative healing was uneventful.

**Figure 7:**
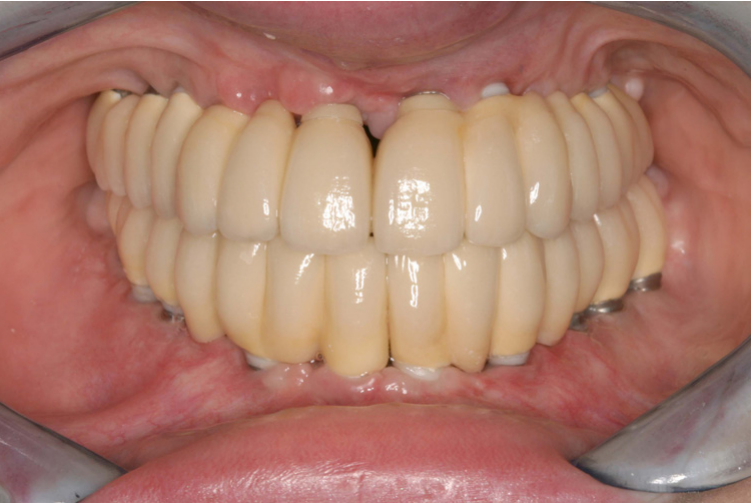
postoperative clinical situation after completion of the implant treatment.

After 4 months of healing, the implants were uncovered and abutment surgery was performed. All implants were completely osseointegrated in the new bone. The patient was provided with a temporary prothesis for two months. After replacing the healing abutments by definite abutments the final restauration was fabricated and inserted (figure 7).

## Discussion

Dentin dysplasia type I is characterized by primary and permanent teeth with normal appearance of the crown but no or only rudimentary root development, incomplete or total obliteration of the pulp chamber and periapical radiolucent areas or cysts [1,2]. The abnormal root morphology is postulated secondary to the abnormal differentiation and/or function of the ectomesenchymally derived odontoblasts [6]. Although various treatment strategies including conventional endodontic therapy, periapical curettage or preventive regimen have been proposed to maintain the teeth as long as possible, early exfoliation of the teeth and maxillomandibular atrophy as a consequence of abnormal root development, periapical abscesses or cystic formations are characteristics of dentin dysplasia type I [7].

Successful oral rehabilitation with complete denture after extraction of all teeth and curettage of cysts has been described [8].

When implant supported prostheses are planned in patients affected by dentin dysplasia type I bone regenerative therapy is required. Munoz-Guerra et al. reported successfull treatment of a 24-year old girl after onlay bone grafting and sinus augmentation [9]. The authors used cortico-cancellous bone blocks from the iliac crest for onlay grafting and and a mixture of autologous bone graft and an autologous platelet concentrate obtained from platelet-rich plasma for the sinus lift procedure. The teeth were extracted 4 months after bone augmentation was performed. No increased and accelerated bone resorption was observed.

In our patient, extraction of all teeth, cystectomy, bilaterally sinus lifting and onlay bone grafting with autogenous bone grafts were performed as the initial surgical procedure. Already 2 months after bone grafting first signs of bone resorption were noted.

Resorption of grafted bone is a well known phenomena that arises during healing and osseointegration processes and as the result of non physiological loading [10]. Bell et al. found a 33% resorption rate of mandibular onlay grafts from the iliac crest during the 4 to 6 months before implant placement. After implant placement resorption rate decreased considerably [11]. Several investigations revealed a high resorption rate of autogenous bone grafts in the period after grafting and before implant placement and therefore recommend a mixture of autogenous bone with allografts [12,13] or stabilizing titanium mesh for vertical alveolar ridge augmentation [14]. Nevertheless the presence of a dehiscence defect irrespective of the augmentation treatment used increases the resorption rate [15]. Bone grafting simultaneous to implant placement has been published to be a proper strategy as this can reduce the number of surgical interventions and additionally fix the implant itself [16]. However a staged procedure is recommended to achieve better implant positioning after graft consolidation. When iliac bone is used, second surgeries may be performed at 4 to 6 months [17]. After an uneventful healing period of 6 month the grafted bone around the implants will have a prognosis similar to that of nongrafted bone [18]. The application of autologous blood plasma enriched with thrombocytes by centrifugal concentration (platelet-rich plasma: PRP) has been accredited to enhance the formation of new bone and improve incorporation and preservation of bone grafts [19]. Platelet-rich plasma (PRP) is being used to deliver growth factors in high concentration to sites requiring osseous grafting. Growth factors released from the platelets include platelet-derived growth factor, transforming growth factor beta, platelet-derived epidermal growth factor, platelet-derived angiogenesis factor, insulin-like growth factor 1, and platelet factor 4. These factors signal the local mesenchymal and epithelial cells to migrate, divide, and increase collagen and matrix synthesis. However there is still lack of scientific evidence to support the effect of PRP on osteogenic induction and the use of PRP in combination with bone grafts during augmentation procedures [20,21]. Although Thor et al. could not demonstrate obvious positive effects of PRP on bone graft healing the authors observed that the handling of the particulated bone grafts was improved [19].

In our patient implant placement was performed as a second stage procedure. A short period after onlay bone grafting and sinus lifting a high degree bone resorption had occurred, although healing was uneventfull and no dehiscence defect had occured. In this situation presurgical implant planning using 3D images (SimPlant^® ^technology) was a helpful tool in this anatomic difficult situation. We were able to take into account not only the present bone volume and morphology but also aesthetic considerations regarding the prosthetic treatment. Implant placement was facilitated by the use of osseous-borne stereolithographic drilling guides. To prevent further extensive secondary bone resorption the principle of guided bone regeneration was used during the second procedure. In the present case, despite the hypothesized increased resorption activity, the secondary performed bone augmentation with a mixture of allogenic materials and autogenous bone in combination with a resorbable membrane provided a successful longterm result. Munoz-Guerra et al. recommend a two stage procedure and the use of autologous cortico-cancellous grafts from the iliac crest for treatment of their patient with dentin dysplasia type I [9]. In contrast to our case Munoz-Guerra et al. did not find an increased affinity for bone resorption in their patient, but they did not perform tooth extraction and cystectomy before bone augmentation but removed the teeth 4 months after onlay bone grafting and sinuslifting was performed. Whether this is the crucial difference in treatment strategy or whether patients afflicted by dentin dysplasia I posses an increased affinity for bone resorption has to be discovered by future research.

## Conclusion

Oral rehabilitation of patients with dentin dysplasia type I requires elaborate treatment planning. Surgical implant planning based on stereolithographic technique is a helpful tool in such cases. As we found an increased affinity for bone resorption in our patient we recommend guided bone regeneration using a decelerated biodegradable collageneous membrane and a mixture of autogenous bone with non resorbable grafting material.
